# Gastric perforation mimicking acute cholecystitis: a case report

**DOI:** 10.1093/jscr/rjag541

**Published:** 2026-06-30

**Authors:** Olivia D Flessland, Matthew Habina, Ryan Lints, Jonathan Levy

**Affiliations:** Department of General Surgery, University of Michigan Health – Sparrow, 1200 E Michigan Ave. #655, Lansing, MI 48912, United States; Department of Trauma and Surgical Critical Care, University of Michigan Health – Sparrow, 215 E. Michigan Ave., Lansing, MI 48912, United States; Michigan State University College of Human Medicine, 965 Wilson Road, Suite A110 (East Fee Hall), East Lansing, MI 48824, United States; Department of General Surgery, University of Michigan Health – Sparrow, 1200 E Michigan Ave. #655, Lansing, MI 48912, United States

**Keywords:** peptic ulcer disease, gastric perforation, cholecystitis

## Abstract

Peptic ulcer disease is a common disorder typically presenting with right upper quadrant and epigastric pain. It can result in perforation when not identified and treated in a timely fashion. We report a case of a 71-year-old female who presented with a workup consistent with acute cholecystitis, who was found to have a gastric ulcer perforation intraoperatively when the prepyloric perforated ulcer was found to be adherent to the gallbladder wall. The case was treated via robotic-assisted laparoscopic cholecystectomy. The cholecystectomy was followed by primary closure of the ulcer defect with omental buttress without modifying port placement or converting to an open procedure. Our case exemplifies how different pathologies can present with similar physical exam findings and imaging, particularly in the upper abdomen. Additionally, this case demonstrates the safety and efficiency of adjustments to intraoperative technique permitted by robotic-assisted laparoscopy when confronted with unexpected findings.

## Introduction

Peptic ulcer disease (PUD) is a common disease of the distal stomach or proximal duodenum that affects 1% of the US population [1] with a lifetime prevalence of 5%–10% in the general population [2]. Common etiologies include *Helicobacter pylori* infection—typically within duodenal ulcers—and prolonged nonsteroidal anti-inflammatory drug use [1]. Pathogenesis includes the inflammation and subsequent disruption of the intestinal mucosa due to chronic inflammation, leading to an imbalance between gastric acid and mucosal protective factors [2]. Although typically responsive to medical management alone, certain complications—including refractory PUD and perforation—require surgical intervention. Perforations can also involve other discrete anatomical structures, depending on the location [3]. We report a unique case of a patient with a perforated gastric ulcer who presented with the findings typical of acute cholecystitis. Both the cholecystectomy and the repair of the gastric perforation were performed via robotic-assisted laparoscopic procedures during the same operation.

## Case report

A 71-year-old female presented to the emergency department (ED) with dull, constant abdominal pain localized to the right upper quadrant (RUQ) and epigastrium, along with intermittent radiation to her right flank for 3 weeks. The pain progressively worsened and was associated with nausea and non-bloody, non-bilious emesis. Of note, she had undergone an uneventful left total hip arthroplasty 1 month prior and was discharged with—and actively taking—aspirin 81 mg twice daily and meloxicam 5 mg daily.

**Figure 1:**
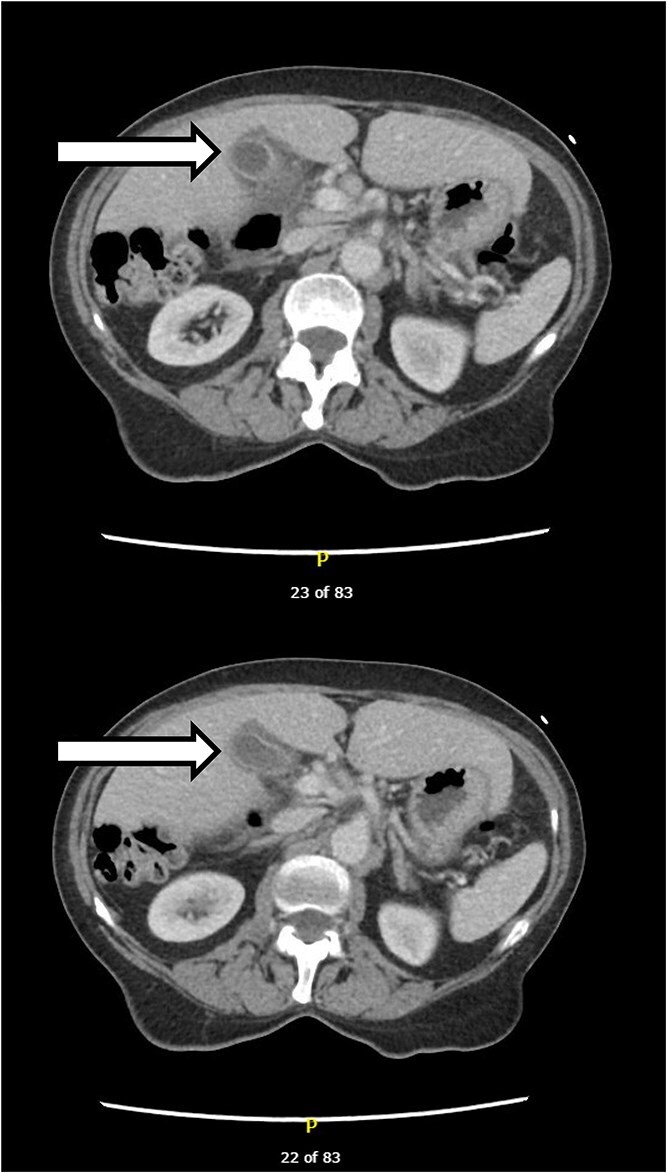
CT findings concerning for acute cholecystitis at the patient’s initial presentation.

**Figure 2:**
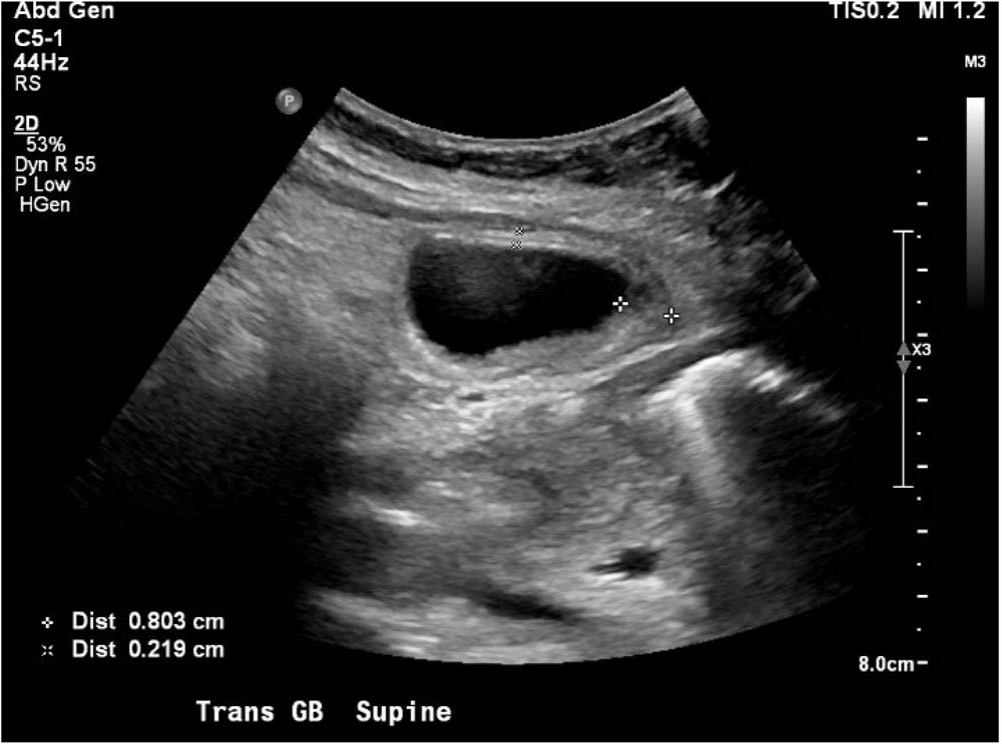
Ultrasound findings concerning for acute cholecystitis when the patient initially presented to the ED.

While in the ED, the patient was found to be afebrile and hemodynamically stable with tenderness in the RUQ and epigastrium. On exam, she was non-peritoneal and had a positive Murphy’s sign. Her labs demonstrated anemia with a hemoglobin of 8.5 g/dl (from 14.3 g/dl two months prior), and normal inflammatory markers. An abdominal and pelvic computed tomography (CT) was obtained, and no evidence of pneumoperitoneum or free fluid was seen. However, there was notable thickening of the gallbladder wall with significant pericholecystic fluid (Fig. 1). Subsequent abdominal ultrasound showed a thickened, edematous gallbladder wall and possible stone in the gallbladder neck (Fig. 2). Given the physical exam and imaging findings, the patient was consented for a robotic-assisted laparoscopic cholecystectomy and a possible intraoperative cholangiogram.

**Figure 3:**
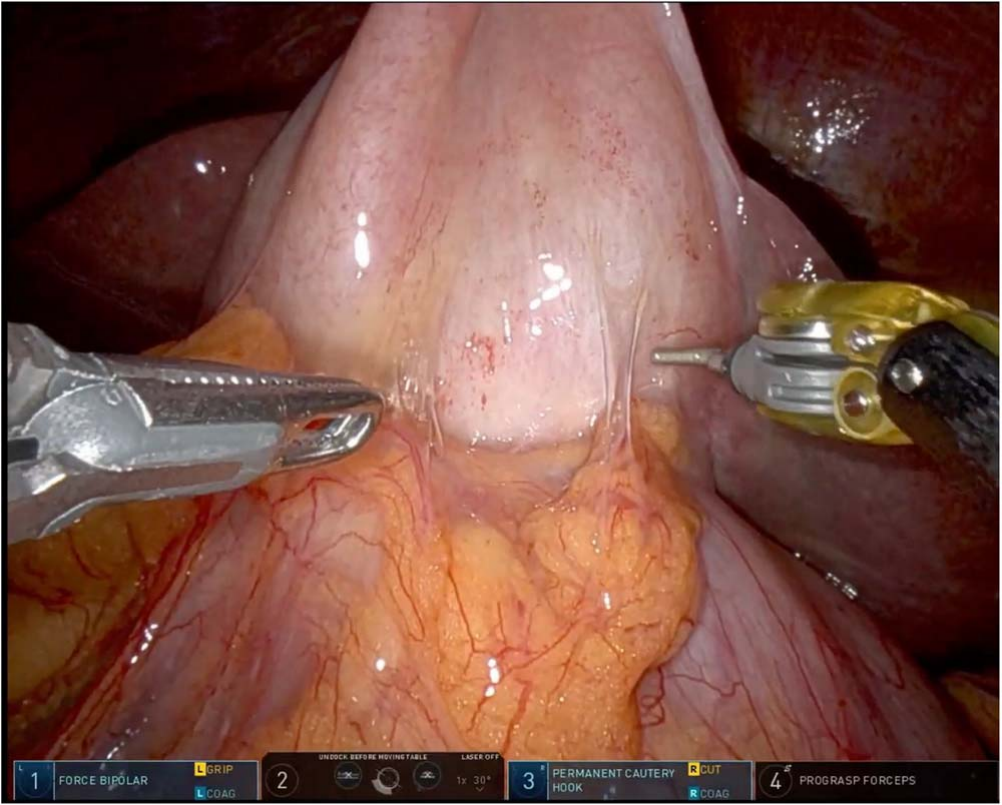
Intraoperative appearance of the gallbladder and adherent stomach with significant adhesions. No obvious surrounding drainage was noted to be draining from around the adherent stomach.

**Figure 4:**
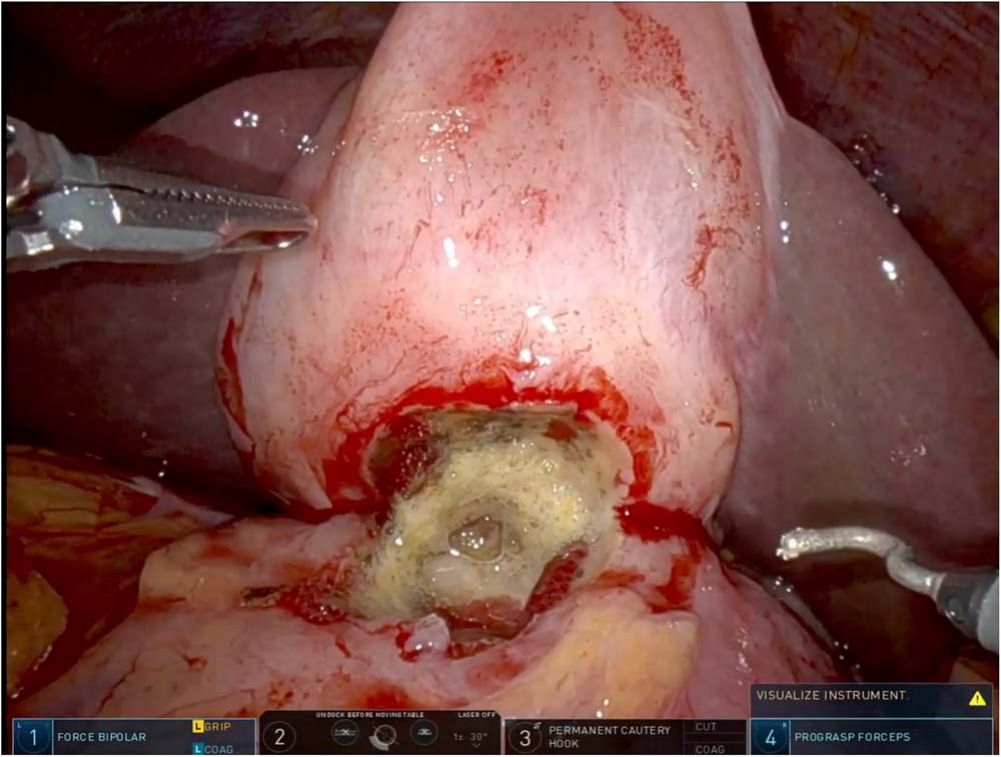
Intraoperative findings of an acutely inflamed gallbladder wall with signs of necrosis where the gastric perforation had sealed itself.

Pneumoperitoneum was achieved with a Veress needle at Palmer point, and an 8 mm port was placed at this location. A 12 mm infraumbilical port was placed, followed by two additional 8 mm ports laterally in the right abdomen. The abdomen was inspected without signs of injury with port placement, and the robot was then docked. The gallbladder was found to be acutely inflamed, with the duodenum and distal stomach adherent to the midportion of the gallbladder (Fig. 3). Using careful blunt dissection, the duodenum and stomach were carefully separated from the gallbladder, revealing a 2 cm prepyloric perforated gastric ulcer causing necrotic changes to the gallbladder wall (Fig. 4). Uncertain of the viability of the gallbladder given its appearance, the cholecystectomy proceeded without issue in the standard fashion. The critical view of safety was obtained and confirmed with Indocyanine Green and Firefly near-fluoroscopy. The gallbladder was removed from the liver after safely clipping and transecting the cystic duct and artery.

**Figure 5:**
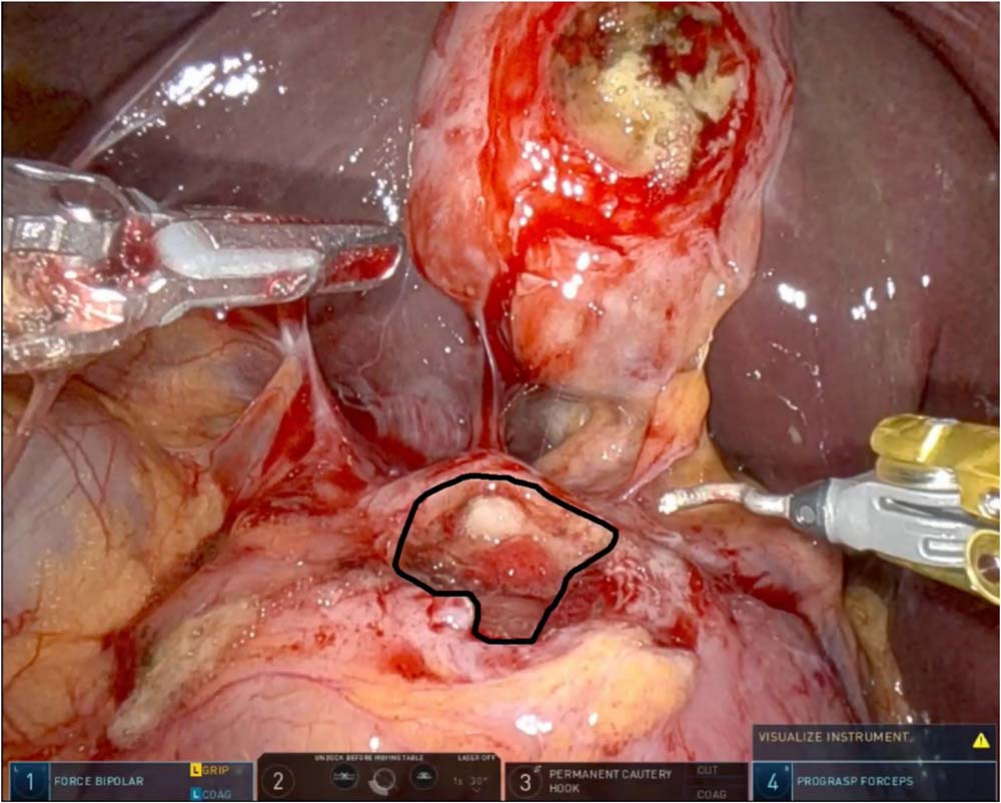
Once the stomach was dissected away from the gallbladder, the perforated ulcer (outlined in black) was more easily assessed and found to contain exposed mucosa and gastric folds, confirming a prepyloric perforated gastric ulcer.

**Figure 6:**
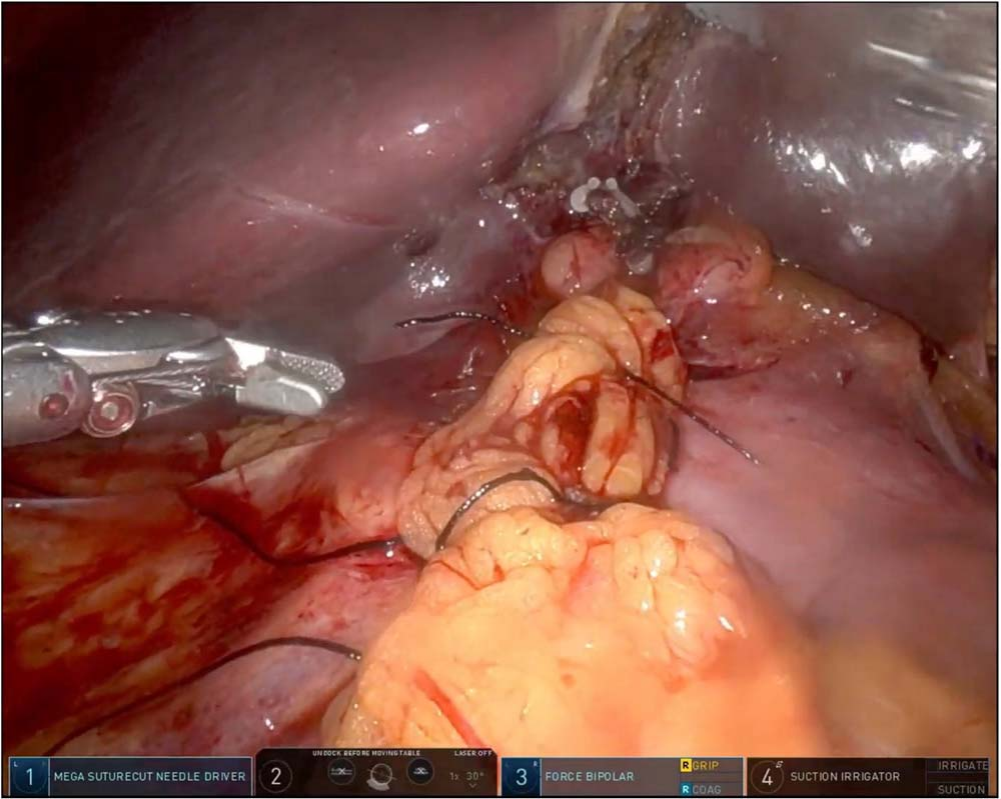
The perforated gastric ulcer repair after the omental buttress was tied into place. At the base of the liver bed, the clips previously applied to the cystic duct are visible.

Attention was turned toward the gastric perforation. Visual assessment of the defect revealed gastric folds within the stomach just proximal to the pylorus, confirming a prepyloric gastric perforation (Fig. 5). Without modifying port placement, the ulcer was closed primarily using interrupted 3-0 silk sutures and subsequent placement of an omental buttress sutured into place (Fig. 6). An intraoperative esophagogastroduodenoscopy was then performed with a negative leak test; additionally, the scope was passed into the duodenum without narrowing of the repair site. Biopsies were deferred at this time due to the friable tissues involved. A Jackson-Pratt drain was left in place at the repair site, the robot was undocked, and the incisions were closed.

Postoperatively, the patient was started on broad-spectrum antibiotics, a prophylactic antifungal, and a proton-pump inhibitor (PPI) twice daily to treat the perforation. An upper GI series was obtained on postoperative Day 2, confirming no contrast extravasation. A negative *H. pylori* fecal antigen was obtained postoperatively. Final gallbladder pathology revealed acute serosal inflammation *without* evidence of acute cholecystitis. The drain was removed on postoperative Day 4. The patient was subsequently discharged home on a PPI twice daily and a short course of oral antibiotics, recovering without incident.

## Discussion

Perforated peptic ulcer disease presenting clinically as acute cholecystitis has been reported in few prior case reports [4, 5]. In this case, the patient developed a perforated gastric ulcer that had sealed itself off via the nearby gallbladder, inducing gallbladder serosal inflammation and thus the clinical picture of acute cholecystitis. Although imaging and physical exam were consistent with acute cholecystitis, the patient’s history and new anemia should have increased suspicion for ongoing PUD. Nevertheless, the patient’s significant pain and chronicity warranted operative intervention.

At our institution, the robotic platform is used increasingly to perform laparoscopic cholecystectomies. By proceeding with robotic-assisted laparoscopy, the superior dexterity of robotic instruments permitted safe surgical intervention to proceed despite the unexpected operative findings, allowing for both the cholecystectomy and gastric ulcer perforation repair without interruption of the procedure. Importantly, this was performed without altering port placements or conversion to an open procedure. This case demonstrates the broad utility of the robotic platform in acute care surgery, even when used for ‘bread and butter’ cases. The robotic option allows safe, efficient adjustments in technique when deviations from anticipated management are required.

## Conclusion

Our case report presents a perforated PUD as an unusual etiology for clinically suspicious acute cholecystitis, leading to unexpected intra-operative findings that were successfully managed via robot-assisted laparoscopic surgery. Even with imaging and physical exam findings consistent with acute cholecystitis, PUD should often remain high in the differential for RUQ pain. Additionally, this case demonstrates the importance of the robotic platform, allowing us to safely and efficiently manage both the cholecystitis and the gastric perforation found intraoperatively, which otherwise may not have been possible using standard laparoscopy.

## References

[1] Vakil N . Peptic ulcer disease: a review. JAMA 2024;332:1832–42. 10.1001/jama.2024.19094 39466269

[2] Lanas A, Chan FKL. Peptic ulcer disease. Lancet 2017;390:613–24. 10.1016/S0140-6736(16)32404-7 28242110

[3] Kavitt RT, Lipowska AM, Anyane-Yeboa A et al. Diagnosis and treatment of peptic ulcer disease. Am J Med 2019;132:447–56. 10.1016/j.amjmed.2018.12.009 30611829

[4] Turner EJ, Owen ER, Reddy K. A 3 cm sealed pyloric perforation discovered during emergency cholecystectomy. BMJ Case Rep 2010;2010:bcr01.2009.1534. 10.1136/bcr.01.2009.1534 PMC3028007 22419948

[5] Konagaya K, Kume N, Ogino H. Duodenal ulcer perforation causing acute cholecystitis. Cureus 2024;16:e61293. 10.7759/cureus.61293 38813075 PMC11134304

